# PROTOCOL: Street outreach conflict mediation programs for reducing violence

**DOI:** 10.1002/cl2.1388

**Published:** 2024-03-03

**Authors:** Edward R. Maguire, Cody W. Telep, Thomas Abt, Ericka Adams

**Affiliations:** ^1^ School of Criminology & Criminal Justice Arizona State University Phoenix Arizona USA; ^2^ Department of Criminology and Criminal Justice University of Maryland College Park Maryland USA; ^3^ Department of Justice Studies San José State University San José California USA

## Abstract

This is a protocol for a Cochrane Review (intervention). The objectives are as follows: This systematic review will synthesize the available evaluation research on the effectiveness of street outreach conflict mediation programs. The review seeks to answer the following primary question: Are street outreach worker strategies that use conflict mediation and/or violence interruption strategies effective at reducing violence? Assuming a sufficient number of eligible studies, this review will also address two additional questions: Are there certain program elements that render these strategies more or less effective? Are there certain conditions under which these strategies are more or less effective? As policymakers struggle to understand the policy options available to them for preventing and reducing violence, having clear answers to these three questions will help them make more informed decisions. The primary focus of this review is the effect of these strategies on violence. Nonetheless, when data are available we will collect information on secondary outcomes such as the cost‐effectiveness of these strategies and their impacts on perceptual or attitudinal measures such as fear, perceived safety, and violence‐related norms.

## 1 BACKGROUND

### 1.1 The problem, condition or issue

The World Health Organization defines community violence as a form of interpersonal violence that “includes youth violence; assault by strangers; violence related to property crimes; and violence in workplaces and other institutions” (World Health Organization n.d., section 2). The U.S. Centers for Disease Control and Prevention define it as violence happening “between unrelated individuals, who may or may not know each other, generally outside the home” (Centers for Disease Control and Prevention, 2022, para. 1). The U.S. Department of Justice defines community violence as “generally happening outside the home in public spaces” (U.S. Department of Justice, 2022, para. 1).

In many countries, community violence accounts for more violent deaths than any other form of crime or violence. It is largely the province of disadvantaged and disenfranchised men who constitute the majority of its perpetrators and victims. Such violence often occurs in urban areas, particularly in a city's most marginalized neighborhoods. It is committed with and without weapons, but the vast majority of community violence homicides involve firearms.

Community violence can occur in the course of crimes such as robbery or kidnaping, but often it is sparked by interpersonal conflicts. According to the Federal Bureau of Investigation's Supplemental Homicide Reports, 64% of all homicides in the United States in 2017 where a motive was identified were the result of disputes of some kind (Abt, 2019). These conflicts often involve rivalries between loosely organized groups often referred to as gangs, cliques, sets, and crews, among other names. Due to the often retaliatory nature of such violence, there is significant overlap between offender and victim populations (Berg & Schrek, 2022; Papachristos et al., 2015; Singer, 1986).

### 1.2 The intervention

In response to rising rates of community violence and to the simultaneous demand for alternatives to traditional law enforcement approaches, a wide array of non‐punitive, community‐led, anti‐violence strategies have been advanced in recent years (Pugilese et al., 2022). These strategies, broadly known as “community violence interventions,” use a wide range of methodologies, but most seek to engage those at the highest risk for violence and provide some form of treatment, support, or services to interrupt ongoing cycles of violence.

Many of these strategies employ street outreach workers to engage high‐risk individuals and groups. Street outreach has a long history (Klein, 1971) and is not limited to violence reduction – outreach workers are often deployed to address other chronic challenges such as homelessness and drug addiction (Decker et al., 2008). Street outreach workers, also known as “credible messengers” or “neighborhood change agents,” typically leverage shared identities and experiences with impacted community members to influence decision making and motivate behavior change.

One type of community violence intervention strategy employs street outreach workers to mediate or interrupt violent or potentially violent conflicts. These programs focus heavily on preventing retaliation, particularly when group‐related acts of violence may result in ongoing cycles of tit‐for‐tat violence. The best‐known example of these programs is *Cure Violence*, though it is not the only initiative in which street outreach workers play a central role in attempting to prevent violence (Butts et al., 2015). The goal of the proposed systematic review is to synthesize the research evidence on those interventions that specifically use conflict mediation and/or violence interruption strategies for reducing violence.

Street outreach conflict mediation programs typically involve assigning outreach workers or “violence interrupters” to work in communities with high rates of violence, acquaint themselves with those likely to be involved in community violence, and engage in a range of activities meant to prevent such violence. The conflict mediation or violence interruption activities may involve, for instance, talking people out of carrying out an imminent act of violence; arranging for influential people (friends, relatives, faith leaders) to convince a likely offender not to carry out an imminent act of violence; transporting a likely victim of violence to a safe place; or arranging a truce among individuals or groups that are likely to commit imminent acts of violence against one another. The specific activities of outreach workers are explicitly separate from law enforcement efforts to reduce violence and generally involve little, if any, direct partnership or communication between outreach workers and police departments. That said, street outreach conflict mediation programs may engage at a more general level with other anti‐violence strategies, including those involving law enforcement.

As noted above, community violence intervention strategies adopt a wide range of approaches to achieve their intended outcomes. These strategies often offer intensive case management, transformational mentoring, subsidized employment, cognitive behavioral therapy, wrap‐around services, and other supports. They may also conduct public awareness campaigns to change community norms concerning violence. Community violence intervention strategies may happen in a wide range of settings, including institutional settings such as schools, hospitals, and juvenile and adult correctional facilities.

This review includes strategies that may use multiple approaches in multiple settings, but included interventions must feature conflict mediation or violence interruption activities that take place in a community setting, rather than an institutional or other setting, as a primary means of achieving the intended outcome of reduced violence. Moreover, they must be *community‐based*. For purposes of this review, interventions that are led by law‐enforcement agencies, schools, hospitals, or correctional agencies will not be considered community‐based and will be excluded. However, otherwise eligible community‐based interventions involving partnerships or collaborations with these and other organizations will be included.

### 1.3 Description of the condition

See previous section.

### 1.4 Description of the intervention

See previous section.

### 1.5 How the intervention might work

Street outreach workers in this context seek to reduce violence by intervening directly with would‐be offenders and their associates to mediate conflicts. Cure Violence, one of the best‐known street outreach conflict mediation programs, relies on three mechanisms for reducing violent behavior. The first involves detecting and interrupting potentially violent conflicts through mediation efforts with offenders, victims, and others who may be able to exert influence. The second involves identifying and treating people at highest risk for behaving violently in an effort to alter their behavior. The third involves engaging with the community in an effort to change community norms about violence. Cure Violence is modeled after public health efforts for reducing disease transmission. Its focus on reducing the transmission of violence is consistent within social science research findings showing how violence – including gang‐related and gun‐related violence – propagates throughout social networks (e.g., Green et al., 2017; Papachristos et al., 2013; Papachristos & Wildeman, 2014). Street outreach worker programs like Cure Violence aim to shape behavior “by relying on the normative power of the social environment rather than on the coercive power of law enforcement and prosecution” (Butts et al., 2015, pp. 48–49). Other street outreach conflict mediation programs use similar but not necessarily the same methodologies.

### 1.6 Why it is important to do this review

For policymakers seeking to reduce gun, group, and gang‐related violence, street outreach conflict mediation programs are one of the most well‐known non‐law enforcement options currently available. These programs have recently received significantly increased attention for at least two reasons. First, in the wake of the COVID‐19 pandemic and social unrest following the murder of George Floyd by a police officer in Minneapolis, the nation has experienced a significant increase in homicides and other forms of community violence. Second and relatedly, demand for non‐police anti‐violence strategies has increased dramatically. Unprecedented amounts of funding are being made available for such strategies at the federal, state, and local levels.

Despite their long history, the evaluation evidence on Cure Violence and similar street outreach initiatives is of variable quality, and the findings vary widely. Some studies have found that such programs reduce violence, others report no effect on violence, and some have found that they increase violence (e.g., see Buggs et al., 2022; Delgado et al., 2018; Fox et al., 2015; Maguire et al., 2018; Picard‐Fritsche & Cerniglia, 2013; Webster et al., 2012, 2013; Whitehill et al., 2013, 2014; Wilson & Chermak, 2011; Wilson et al., 2010). This variability in both the quality and outcomes leaves policymakers in a difficult position, where they are unable to make sense of a conflicting body of research evidence. The proposed systematic review seeks to make sense of this growing body of research by synthesizing the highest‐quality research evidence on whether these types of initiatives are effective in reducing violence.

## 2 OBJECTIVES

This systematic review will synthesize the available evaluation research on the effectiveness of street outreach conflict mediation programs. The review seeks to answer the following primary question:

- Are street outreach worker strategies that use conflict mediation and/or violence interruption strategies effective at reducing violence?

Assuming a sufficient number of eligible studies, this review will also address two additional questions:

- Are there certain program elements that render these strategies more or less effective?
- Are there certain conditions under which these strategies are more or less effective?

As policymakers struggle to understand the policy options available to them for preventing and reducing violence, having clear answers to these three questions will help them make more informed decisions.

The primary focus of this review is the effect of these strategies on violence. Nonetheless, when data are available we will collect information on secondary outcomes such as the cost‐effectiveness of these strategies and their impacts on perceptual or attitudinal measures such as fear, perceived safety, and violence‐related norms.

## 3 METHODS

### 3.1 Criteria for considering studies for this review

#### 3.1.1 Types of studies

To be eligible for inclusion in this systematic review, studies must contain one or more quantitative estimates of the impact of street outreach worker programs on a measure of the incidence or prevalence of violence. Eligible studies are those that rely on experimental designs that randomize geographic areas or individuals to intervention or comparison conditions, or quasi‐experimental designs that include comparison areas/groups that have not received the intervention. While experimental designs often provide the strongest basis for drawing inferences about the efficacy of an intervention, we are not familiar at this stage of the process with any such studies on the effects of street outreach worker programs. We agree with Wilson et al. (2023, p. 7), who caution against “restricting study inclusion to a single purportedly high‐quality design” in all but the rarest of circumstances.

Thus, limiting this review to experimental studies would likely result in either no, or a very small number of, eligible studies. The term “quasi‐experimental designs” is broad and includes a wide range of designs. In this study, we are guided in part by the types of randomized and quasi‐experimental studies identified by Wilson et al. (2023). Eligible studies will employ one of the following types of designs. All will include a comparison group (with the exception of sufficiently lengthy interrupted time series studies):

- Randomized controlled trial (RCT), where geographic areas or individuals are randomly assigned to receive an intervention (street outreach program) or control condition
- Non‐equivalent groups design, pretest/posttest
- Non‐equivalent groups design, pretest/posttest, with baseline measures (difference‐in‐difference analysis)
- Non‐equivalent groups design, pretest/posttest with matching on observed characteristics (including propensity score matching)
- Regression discontinuity design
- Instrumental variable analysis
- Interrupted time‐series design without a comparison series, but where there are at least 24 data points in the pre and post‐intervention period (see Lum et al., 2020)
- Interrupted time‐series designs with comparison series
- Synthetic control design

We will exclude single group designs with single pre‐ and post‐intervention measures. That is, studies that evaluate a street outreach worker program without some sort of comparison area/group will not be included.

#### 3.1.2 Types of participants

We anticipate that the most frequently used units of analysis in the studies included in this review will be relatively small geographic areas such as neighborhoods and communities. We will not consider larger geographic units such as nations, subnational regions, states, or counties for inclusion since such units do not align with the focused nature of the intervention on concentrations of violence. Otherwise, we do not envision imposing other restrictions on geographic units of analysis.

We also acknowledge the possibility that individuals deemed to be at risk for carrying out violence or being victimized by it could serve as the unit of analysis in some studies. If this is true, we will meta‐analyze the area‐based and individual‐based studies separately.

#### 3.1.3 Types of interventions

Our specific interest is in community‐based street outreach worker programs that use conflict mediation or violence interruption strategies to reduce violence in a community setting, with a focus on specific geographic areas and/or individuals. Please see the “Background” section above for more detailed information.

#### 3.1.4 Types of outcome measures

We describe our primary and secondary outcomes in the sections below.

##### Primary outcomes

The principal outcomes of interest for area‐based studies are measures of violence at the neighborhood or community level. The measures of violence used in these studies are likely to differ depending on the geographic location and specific aim of each individual initiative. The most typical measures are homicides, shootings, serious assaults/woundings, hospital gunshot wound admissions, and calls to the police for violent incidents. To be eligible for inclusion, area‐based studies must contain one or more measures of the incidence or prevalence of violence.

For individual‐based studies, measures of violent offending could be based on self‐reports and/or official data from police/criminal justice agencies. Measures of violent victimization could be based on a variety of data sources, including surveys, official police/criminal justice data, or hospital data. In either case, these measures will focus on serious acts of violence including assaults, shootings, stabbings, attempted murders, and murders.

##### Secondary outcomes

Some studies also contain measures of secondary outcomes derived from survey data. Such measures include outcomes like fear of crime and social norms conducive to the use of violence. To the extent that such measures are available, we will include them as secondary outcomes.

### 3.2 Search methods for identification of studies

To identify studies eligible for this systematic review, we will rely on six search strategies. First, we will search electronic databases in social sciences and public health (see below for additional details). Second, we will search the bibliographies of books, chapters, articles, and reports that contain discussions of street outreach worker initiatives. These will include, for example, Butts et al. (2015), Ransford et al. (2019), Skogan et al. (2009), Slutkin (2013), Webster et al. (2013), and Wilson and Chermak (2011). We will identify any eligible studies from these existing relevant reviews. Third, we will contact leading researchers in the violence prevention space, including criminologists, public health scholars, and scholars from other disciplines who specialize in community violence prevention to identify eligible studies that are not yet published or publicly available. Fourth, we will contact people involved in administering street outreach worker initiatives in an effort to identify unpublished evaluations. Fifth, we will review the bibliographies of all identified eligible studies to look for any additional relevant studies. Sixth, we will conduct forward citation searches for both the key studies and reviews noted in our second search strategy, as well as all eligible studies. We will use the “cited by” feature in Google Scholar to conduct these forward citation searches. This systematic review will be based on an exhaustive search that seeks to identify all eligible studies whether published or unpublished.

#### 3.2.1 Electronic searches

The following databases will be searched:

1. Academic Search Premier (via EBSCOhost)
2. Book Citation Index – Social Sciences and Humanities (via Web of Science)
3. CINAHL Plus (via EBSCOhost)
4. CINCH: Australian Criminology Database (via Australian Institute of Criminology)
5. Conference Proceedings Index: Social Science and Humanities (via Web of Science)
6. Criminal Justice Abstracts (via EBSCO host)
7. Dissertations & Theses Global (via ProQuest)
8. EconLit (via EBSCO host)
9. Education Resources Information Clearinghouse (ERIC) (via ProQuest)
10. GeoRef (via GeoScienceWorld)
11. JSTOR
12. LexisNexis Academic/NexisUni (via LexisNexis)
13. MedLine (via ProQuest)
14. National Criminal Justice Reference Services (NCJRS) (via EBSCOhost)
15. PAIS International (via ProQuest)
16. PsycINFO (via ProQuest)
17. PubMed (via National Library of Medicine)
18. Rutgers School of Law‐Newark Gray Literature Database
19. Social Science CitationIndex (via Web of Knowledge)
20. Social Science ResearchNetwork (SSRN)
21. Social Services Abstracts (via ProQuest)
22. Sociological Abstracts (via ProQuest)
23. SocINDEX (via EBSCOhost)
24. WorldCat (via OCLC)

The following trial registries will be searched:

- Australian and New Zealand Clinical Trials Registry
- ClinicalTrials.gov
- Clinical Trials Results
- Cochrane Central Register of Controlled Trials (CENTRAL)
- ISRCTN Registry (controlled-trials.com)
- NIH RePORTER
- Trials Register of Promoting Health Interventions (TRoPHI)
- Unreported Trials Register
- UK Clinical Research Network (UKCRN Study Portfolio)
- WHO International Clinical Trials Registry Platform

The publications of the following groups will also be searched:

- Advance Peace (https://www.advancepeace.org/about/learning-evaluation-impact/)
- Center for CourtInnovation (https://www.courtinnovation.org/publications?keys=&aof=All&program=&page=0)
- Cure Violence (https://cvg.org/impact/#EvidenceSummary)
- Everytown for Gun Safety (https://everytownresearch.org/research/)
- Giffords Law Center (https://giffords.org/lawcenter/resources/reports/)
- Institute for Law and Justice (https://ilj.org/publications/index.html)
- Inter‐American Development Bank (https://www.iadb.org/en/research-and-data/publications)
- John Jay College of Criminal Justice Research and Evaluation Center (https://johnjayrec.nyc/recbibliography/)
- Johns Hopkins Center for Injury Research and Policy (https://www.jhsph.edu/research/centers-and-institutes/johns-hopkins-center-for-injury-research-and-policy/resources-library/)
- Johns Hopkins Gun Violence Solutions (https://publichealth.jhu.edu/departments/health-policy-and-management/research-and-practice/center-for-gun-violence-solutions)
- Justice Research and Statistics Association – State Statistical Analysis Centers (SACs) Publication Library (https://justiceresearch.dspacedirect.org/collections/a618488f-41a9-468b-afa0-782c4ccd7345?cp.page=1)
- National Policing Institute (https://www.policinginstitute.org/publications/)
- Police Executive Research Forum (https://www.policeforum.org/free-online-documents)
- The Police Foundation (https://www.police-foundation.org.uk/publications/#1515760959315-3732af47-a460)
- Rand Corporation (https://www.rand.org/pubs.html)
- Urban Institute (https://www.urban.org/research-area/crime-justice-and-safety)
- Urban Peace Institute (https://www.urbanpeaceinstitute.org/publications)
- U.S. Department of Health and Human Services (https://www.cdc.gov/violenceprevention/index.html)
- U.S. Department of Justice (http://www.ojp.gov)
- Vera Institute for Justice (https://www.vera.org/solutions-research)
- World Bank (https://www.worldbank.org/en/research)
- World Health Organization (https://www.who.int/publications/i)

The keywords in Table 1 will be used to search the databases listed above. Searches will be conducted in English, Spanish, and Portuguese. There are no date limitations to the search. We will search all terms across title, abstract, author‐supplied, keywords, and indexing terms. Terms for the intervention (Concept 1), outcome (Concept 2) and evaluation method (Concept 3) will be used to ensure a search broad enough to identify all eligible studies and narrow enough to minimize irrelevant hits. For each database, our searches will cover (Concept 1) AND (Concept 2) AND (Concept 3). Example search syntax is provided as a Supporting Information: Appendix to this protocol.

**Table 1:** Search terms.

Concept 1 (intervention)	Concept 2 (outcome)	Concept 3 (evaluation)
“Advance Peace” Ceasefire “Cease Fire” “credible messenger” CureViolence “Cure Violence” (community NEAR/3 outreach*) (conflict* NEAR/3 mediat*) (conflict* NEAR/3 resol*) (gang NEAR/3 interven) (gang NEAR/3 mediat*) (gang NEAR/3 outreach*) outreach* peacemak* (street* NEAR/3 interven*) (street* NEAR/3 mediat*) (street* NEAR/3 program*) (street* NEAR/3 work*) truce* (violen* NEAR/3 interrupt*) (violen* NEAR/3 interven*) (violen* NEAR/3 program*)	assault* attack* death* “gun” “guns*” homicide* lethal* kill* manslaughter murder* shoot* “stab” stabbing* violen* weapon*	effective* efficac* evaluat* experiment* interven* quasi‐experiment* “quasi experiment*” random* RCT trial* “what works*”

#### 3.2.2 Searching other resources

Our second, third, fourth, fifth, and sixth search strategies (reviewing bibliographies of seminal works, contacting researchers in the field, contacting practitioners in the field, forward searches of seminal works and eligible studies, and reviewing bibliographies of eligible studies) will all involve searching other resources in an effort to identify the universe of available studies.

### 3.3 Data collection and analysis

#### 3.3.1 Description of methods used in primary research

The studies included in this review will use methodologies that are variations of a treatment versus comparison group research design with at least one post‐test measure. Specifically, this review will include randomized experimental studies and a variety of types of quasi‐experimental studies as described in Section 3.1.1.

#### 3.3.2 Criteria for determination of independent findings

We expect studies will report multiple outcomes. We will address such situations so that analyses will not include dependent outcomes in the same analysis. For example, some studies may report on multiple violence outcomes in the same intervention. For cases such as this with multiple findings from the same sample, each will be examined independently to decide how to either combine the findings or to choose the one that best represents the study.

In the case of a single study with multiple sites (e.g., communities or neighborhoods) within the same jurisdiction, and reliant on the same intervention, the results will be treated as multiple outcomes in the same study and will be reported using robust variance estimation procedures designed for handling dependent effect sizes (Hedges et al., 2010; Pustejovsky & Tipton, 2022; Tipton & Pustejovsky, 2015). These analyses will be carried out in R using the “robumeta” package (Fisher & Tipton, 2015). In the case of a single study with multiple sites in different jurisdictions (e.g., a multi‐site intervention), each will be treated as an independent study.

In the case in which study authors identify multiple key outcomes of the study, we will code all these primary outcomes identified by authors, and will report findings using the same robust variance estimation procedures. The same strategy will be used for any studies reporting the same outcome multiple times with different types of data (i.e., a study evaluating the impact of a program on violence that reports on both calls for service and incidents).

#### 3.3.3 Selection of studies

Research assistants will use Zotero to help organize and screen potentially eligible studies. At least two members of the research team will independently screen search results and abstracts from electronic searches to initially assess eligibility based on inclusion criteria. When there is disagreement about eligibility, the review authors will meet to make a final determination. For potentially eligible studies, several strategies will be used to obtain full‐text versions of the studies found through searches of the various abstract databases listed above. First, we will attempt to obtain full‐text versions from the electronic journals available through Arizona State University and the University of Maryland's library research databases. When electronic versions are not available, we will use print versions of journals available at the libraries. If the journals or books are not available, we will make use of the Interlibrary Loan Office (ILL) to try to obtain the journal from the libraries of other universities. If these methods do not work, we will contact the author(s) of the article and/or the agency that funded the research to try to get a copy of the full‐text version of the study.

#### 3.3.4 Data extraction and management

We developed a standardized coding sheet (attached to this protocol as a Supporting Information: Appendix) and will train research assistants on how to use it. In addition to the coding sheet, we will develop a document containing detailed instructions about the coding process. The research assistants will use this coding sheet to code any studies determined to be potentially eligible for inclusion in the systematic review. The coding sheet will be implemented in a Qualtrics data entry sheet to facilitate the data entry process in a user‐friendly format. Once data are entered, they will be exported to the various types of statistical software used by the research team for cleaning and analysis.

The coding sheet captures a variety of information about the study, including a formal APA (7th edition) citation, eligibility criteria for inclusion in the systematic review, the time and location when/where the study was carried out, the quality of the design and analysis, measures of study outcomes, and any additional open‐ended text about the study, its findings, and its quality that are relevant for the systematic review.

All eligible studies will be coded on a variety of criteria including:

1. Reference information (title, authors, publication, etc.)
2. Location of program
3. Nature and description of selection of target site(s)/groups
4. Nature and description of selection of comparison site(s)/groups
5. The unit of analysis
6. The sample size (geographic and/or individual)
7. Methodological type (randomized experiment or quasi‐experiment)
8. A description of the street outreach program and activities that occurred during the intervention
9. Dosage intensity and type
10. Implementation difficulties
11. The statistical test(s) used
12. Statistical diagnostics used (if any)
13. Reports of statistical significance (if any)
14. Effect size (if any)
15. The conclusions drawn by the authors

Two members of the research team will independently code each eligible study. Where there are discrepancies, the lead authors will review the study, discuss the coding decisions with the original coders and determine the final coding decision.

#### 3.3.5 Assessment of risk of bias in included studies

Viswanathan et al. (2018), define risk of bias as “the likelihood of inaccuracy in the estimate of causal effect in that study” (p. 3). We will use the risk of bias tool developed by Lum et al. (2020) in a Campbell Collaboration review of the effects of Body‐Worn cameras. That tool was adapted from the Cochrane risk of bias tool study (Sterne et al., 2019). It is based on a slightly modified Evidence Project's risk of bias tool to assess the risk of bias in the studies included in the systematic review (Kennedy et al., 2019).

#### 3.3.6 Measures of treatment effect

Since the outcome variables in the area‐based studies are continuous count variables, we will calculate and report incident rate ratios (or relative incident rate ratios) for each included analysis (see Spittal et al., 2015; Wilson, 2022). We will also report the 95% confidence intervals associated with each effect size estimate. If there are primary or secondary outcomes reported at the individual level, we will use Cohen's *d* as an effect size estimate.

#### 3.3.7 Unit of analysis issues

The primary unit of analysis in the area‐based studies included in this review will be communities or neighborhoods, and data on crime will typically be reported at the community or neighborhood level. Given that clustered designs or evaluations examining individuals nested within places are not commonly used for street outreach worker programs, we do not anticipate any unit of analysis issues to arise. But if we do find studies that use such designs, we will adjust standard errors as needed to account for clustering.

#### 3.3.8 Dealing with missing data

During the coding process, we will contact authors of original studies in cases where missing or incomplete information makes it difficult to code a study. During the analysis process, we will rely on statistical methods for handling missing data. These methods depend heavily on the mechanism responsible for producing the missing data. If we discover missing data in this systematic review, we will begin by contacting the study authors to obtain any missing data. If that approach is unsuccessful, we will assess the likely causes of any missing data and follow standard statistical approaches for addressing those issues. With a sufficient number of effect sizes, this could include multiple imputation or full‐information maximum likelihood estimation to generate estimates from models in which data are missing for moderator variables (not for effect size estimates). We will be explicit and transparent in reporting any such analyses, and we will conduct sensitivity analyses to assess the extent to which our findings may have changed due to the use of missing data handling procedures.

#### 3.3.9 Assessment of heterogeneity

We will examine the *Q*‐statistic in meta‐analyses to assess heterogeneity of effect sizes across studies. It is our initial assumption that effect size is a random factor in our analysis, and we will plan to implement a random effects model for all analyses involving effect sizes. This is the case because there is diversity in street outreach worker programs. While they share common characteristics, there are likely to be important differences in the exact treatment delivered in eligible studies. With regard to heterogeneity of effects, we will report *τ* ^2^, which “an estimate of the variance in the underlying distribution of true effect sizes” (see van Lissa, 2019, section 7.1). We will also report *I* ^2^, keeping in mind the methodological limitations of that statistic (e.g., Migliavaca et al., 2022; Thorlund, et al., 2012; von Hippel, 2015). All of these measures (the *Q*‐statistic, *τ* ^2^, and *I* ^2^) have strengths and weaknesses that must be considered when assessing heterogeneity, therefore we will report and discuss all of them (van Lissa, 2019).

#### 3.3.10 Assessment of reporting biases

Publication bias is a concern in every meta‐analysis. We plan to use traditional methods to test for the sensitivity of the findings to publication bias in the experimental and quasi‐experimental studies, including the funnel plot and Egger's test (see Sterne et al., 2005; Sterne & Egger, 2005). These methods will include a comparison of the mean effect size for published and unpublished studies. If we discover evidence of publication bias, we will use a trim‐and‐fill analysis to adjust for the number of studies that are missing due to that bias (Duval, 2005).

#### 3.3.11 Data synthesis

Meta‐analytic procedures will be used to combine data from studies. For eligible studies with sufficient data, effect sizes will be calculated using the standardized measures of effect sizes as suggested in the meta‐analytic literature (e.g., see Lipsey & Wilson, 2001). Mean effect sizes will be computed across studies. We will fit a random effects model using a restricted maximum‐likelihood estimator available in the “metafor” package in *R* (van Lissa, 2019; also see Veroniki et al., 2016). If we locate both area‐level and individual‐level studies, we will analyze the two groups of studies separately.

#### 3.3.12 Subgroup analysis and investigation of heterogeneity

We also hope to examine contextual or moderating features of street outreach programs (e.g., based on program location, program features, and evaluation methodology). If we identify enough relevant studies for statistical analysis, we will use the analog to the ANOVA method of moderator analysis (see Lipsey & Wilson, 2001) for categorical moderator variables and meta‐analytic regression analysis for continuous moderator variables or analyses involving multiple moderators. We envision conducting three types of moderator analyses in this study if data permit us to do so. First, if applicable, we will test for the difference between studies using randomized versus non‐randomized designs. Second, if applicable, we will test the difference between studies conducted in different nations and regions. Third, if applicable, we will test for the difference between studies containing different program elements.

#### 3.3.13 Sensitivity analysis

##### Treatment of qualitative research

We do not plan to include qualitative research.

#### 3.3.14 Summary of findings and assessment of the certainty of the evidence

This will be added upon completion of the review.

## CONTRIBUTIONS OF AUTHORS

Content: Ed Maguire, Thomas Abt, and Ericka Adams have content expertise on street outreach conflict mediation programs. Ed Maguire is a criminologist who specializes in policing and violence. He led an evaluation of a street outreach conflict mediation program in Trinidad and Tobago. Thomas Abt is the Founding Director of the Center for the Study and Practice of Violence Reduction and has extensive experience in the research and policy worlds on issues related to community violence. He discusses street outreach worker programs in his well‐known book, *Bleeding Out: The Devastating Consequences of Urban Violence – And a Bold New Plan for Peace in the Streets* and as chair of the Council on Criminal Justice's Violent Crime Working Group. Ericka Adams was part of the research team that evaluated a street outreach conflict mediation program in Trinidad and Tobago. She has published several peer‐reviewed articles from that study. More generally, she specializes in qualitative analysis and the experiences and perspectives of residents who live in communities with high rates of violence.

Systematic review methods: Ed Maguire, Cody Telep, and Thomas Abt, have previous experience with systematic review methodology. Ed Maguire was part of a research team that conducted two meta‐analyses on toxicology study findings among homicide victims. Cody Telep is a criminologist who specializes in policing and experimental methodologies. He has worked previously on Campbell Crime and Justice systematic reviews and meta‐analyses on problem‐oriented policing, displacement in large geographic areas, police patrol, and community policing. Thomas Abt completed a systematic review of community violence reduction efforts for the United States Agency for International Development.

Statistical analysis: Ed Maguire and Cody Telep have extensive experience with a variety of multivariate statistical methods. They will take the lead on all statistical analyses for this systematic review.

Information retrieval: Ed Maguire, Cody Telep, and Thomas Abt have experience with information retrieval methods based on their previous systematic reviews and meta‐analyses. Ed Maguire and Cody Telep will take the lead on the information retrieval portion of the study. Thomas Abt will use his extensive network of contacts in the policy domain to secure unpublished reports containing evaluation findings about street outreach worker programs. The authors will be assisted by a team of research assistants from the School of Criminology & Criminal Justice at Arizona State University. Cody Telep is the Associate Director of the School, and he will ensure that there are enough research assistants to support the information retrieval and coding portions of this project.

## DECLARATIONS OF INTEREST

- Ed Maguire conducted an evaluation of Cure Violence in Trinidad and Tobago.
- Cody Telep has not been involved in the development or evaluation of street outreach worker programs.
- Thomas Abt led the development of New York's GIVE (Gun‐Involved Violence Elimination) Initiative as former Deputy Secretary of Public Safety. Initiatives funded under GIVE included street outreach worker programs. Abt also had an oversight role for the SNUG initiative, which funds street outreach programs throughout the state.
- Ericka Adams conducted an evaluation of Cure Violence in Trinidad and Tobago.

### Preliminary timeframe

We plan to complete this review by October 1, 2023.

### Plans for updating this review

The authors will continue to monitor new research evidence on street outreach worker programs and update this systematic review when a sufficient body of new research evidence is available.

## SOURCES OF SUPPORT

### Internal sources

- Center for the Study and Practice of Violence Reduction at the University of Maryland, College Park, USA, USA

We are grateful to the Center for the Study and Practice of Violence Reduction at the University of Maryland for providingresearch assistants and administrative support for this systematic review.

- Department of Criminology and Criminal Justice at the University of Maryland, College Park, USA

We are grateful to the Department of Criminology and Criminal Justice at the University of Maryland for providing administrative support for this systematic review.

- School of Criminology and Criminal Justice, Arizona State University, USA

We are grateful to the School of Criminology and Criminal Justice at Arizona State University for providing researchassistants to supportthis systematic review.

### External sources

Arnold Ventures, USA

We are grateful to Arnold Ventures for supporting this systematic review.

## Supporting information

Supporting information.
