# Supplementary material for: PROTOCOL: Street outreach conflict mediation programs for reducing violence
Source: Campbell Syst Rev. 2024 Mar 3;20(2):e1388. doi: 10.1002/cl2.1388 (PMC10909390; doi:10.1002/cl2.1388)
Supplement: Supplementary file 1 — Supporting information. [file CL2-20-e1388-s001.docx]

# Appendices

## Appendix 1. Sample Search Syntax for Criminal Justice Abstracts (via EBSCO)

Syntax Terms: TI = Title, AB = Abstract, KW = Keyword, SU = Subject Terms

**Search 1:**

1. TI (“Advance Peace” OR Ceasefire OR “Cease Fire” OR “credible messenger” OR CureViolence OR “Cure Violence” OR (community N3 outreach*) OR (conflict* N3 mediat*) OR (conflict* N3 resol*) OR (gang N3 interven*) OR (gang N3 mediat*) OR (gang N3 outreach*) OR outreach* OR peacemak* OR (street* N3 interven*) OR (street* N3 mediat*) or (street* N3 program*) OR (street* N3 work*) OR truce* OR (violen* N3 interrupt*) OR (violen* N3 interven*) OR (violen* N3 program*) ) OR AB ( (“Advance Peace” OR Ceasefire OR “Cease Fire” OR “credible messenger” OR CureViolence OR “Cure Violence” OR (community N3 outreach*) OR (conflict* N3 mediat*) OR (conflict* N3 resol*) OR (gang N3 interven*) OR (gang N3 mediat*) OR (gang N3 outreach*) OR outreach* OR peacemak* OR (street* N3 interven*) OR (street* N3 mediat*) or (street* N3 program*) OR (street* N3 work*) OR truce* OR (violen* N3 interrupt*) OR (violen* N3 interven*) OR (violen* N3 program*) ) OR KW (“Advance Peace” OR Ceasefire OR “Cease Fire” OR “credible messenger” OR CureViolence OR “Cure Violence” OR (community N3 outreach*) OR (conflict* N3 mediat*) OR (conflict* N3 resol*) OR (gang N3 interven*) OR (gang N3 mediat*) OR (gang N3 outreach*) OR outreach* OR peacemak* OR (street* N3 interven*) OR (street* N3 mediat*) or (street* N3 program*) OR (street* N3 work*) OR truce* OR (violen* N3 interrupt*) OR (violen* N3 interven*) OR (violen* N3 program*) ) OR SU (“Advance Peace” OR Ceasefire OR “Cease Fire” OR “credible messenger” OR CureViolence OR “Cure Violence” OR (community N3 outreach*) OR (conflict* N3 mediat*) OR (conflict* N3 resol*) OR (gang N3 interven*) OR (gang N3 mediat*) OR (gang N3 outreach*) OR outreach* OR peacemak* OR (street* N3 interven*) OR (street* N3 mediat*) or (street* N3 program*) OR (street* N3 work*) OR truce* OR (violen* N3 interrupt*) OR (violen* N3 interven*) OR (violen* N3 program*) )

**Search 2:**

2. TI (murder* OR manslaughter* OR assault* OR violen* OR kill* OR attack* OR homicide* OR shoot* OR “stab” OR stabbing* OR death* OR lethal* OR “gun” OR “guns” OR weapon*) ) OR AB (murder* OR manslaughter* OR assault* OR violen* OR kill* OR attack* OR homicide* OR shoot* OR “stab” OR stabbing* OR death* OR lethal* OR “gun” OR “guns” OR weapon*) ) OR KW (murder* OR manslaughter* OR assault* OR violen* OR kill* OR attack* OR homicide* OR shoot* OR “stab” OR stabbing* OR death* OR lethal* OR “gun” OR “guns” OR weapon*) ) OR SU (murder* OR manslaughter* OR assault* OR violen* OR kill* OR attack* OR homicide* OR shoot* OR “stab” OR stabbing* OR death* OR lethal* OR “gun” OR “guns” OR weapon*) )

**Search 3:**

3. TI (effective* OR efficac* OR evaluat* OR experiment* OR interven* OR quasi-experiment* OR “quasi-experiment” OR random* OR RCT OR trial* OR “what works”) OR AB (effective* OR efficac* OR evaluat* OR experiment* OR interven* OR quasi-experiment* OR “quasi-experiment” OR random* OR RCT OR trial* OR “what works”) ) OR KW (effective* OR efficac* OR evaluat* OR experiment* OR interven* OR quasi-experiment* OR “quasi-experiment” OR random* OR RCT OR trial* OR “what works”) ) OR SU (effective* OR efficac* OR evaluat* OR experiment* OR interven* OR quasi-experiment* OR “quasi-experiment” OR random* OR RCT OR trial* OR “what works”))

**Search 4: (all three searches combined, final search)**

4. “1 AND 2 AND 3”
